# Comparison of Accuracy of Patient and Physician Scar Length Estimates Before Mohs Micrographic Surgery for Facial Skin Cancers

**DOI:** 10.1001/jamanetworkopen.2020.0725

**Published:** 2020-03-11

**Authors:** William C. Fix, Christopher J. Miller, Jeremy R. Etzkorn, Thuzar M. Shin, Nicole Howe, Joseph F. Sobanko

**Affiliations:** 1 Medical student at time of writing, Perelman School of Medicine, University of Pennsylvania, Philadelphia; 2 Department of Dermatology, University of Pennsylvania Health System, Philadelphia

## Abstract

**Importance:**

Patients are satisfied when surgical outcomes meet their expectations. Dissatisfaction with surgical scars is one of the most common reasons that patients sue surgeons who perform Mohs micrographic surgery (MMS).

**Objective:**

To measure the accuracy of patient and physician estimations of scar length prior to skin cancer removal with MMS.

**Design, Setting, and Participants:**

This cross-sectional study was conducted between December 1, 2017, and February 28, 2018, at the MMS clinic of a single tertiary referral center health system. A total of 101 adults presenting for MMS for treatment of facial skin cancers volunteered for this study, and 86 surgeons who performed the MMS procedure participated.

**Main Outcomes and Measures:**

Patients and physicians independently drew the anticipated scar length on the patients’ skin prior to surgery. Preoperative estimates by patients and surgeons were compared with actual postoperative scar length.

**Results:**

Of the 101 patients who participated, 57 patients (56.4%) were men and 57 patients (56.4%) were aged 65 years or older. Eighty-four patients (83.2%) underestimated scar length, whereas 67 of the 86 surgeons (77.9%) correctly estimated the scar length (*P* < .001). The actual postoperative scar length was 2.2 (interquartile range, 1.5-3.6) times larger than the patients’ preoperative estimate but only 1.1 (interquartile range, 1.0-1.2) times larger than the surgeons’ preoperative estimate (*P* < .001). Preoperative consultation with the surgeon, a personal history of MMS, or patient-directed research about MMS were not associated with improvement of patients’ estimations of scar length.

**Conclusions and Relevance:**

This study’s findings suggest that patients with facial skin cancers have unrealistic expectations regarding scars that measure, on average, less than half the length of the actual postoperative scars. Surgeons appear to accurately estimate the length of most surgical scars and have an opportunity to set realistic patient expectations about scar length before surgery.

## Key Points

**Question:**

How accurate are patients’ and surgeons’ preoperative estimates of the final scar length from Mohs micrographic surgery for facial skin cancers?

**Findings:**

In this cross-sectional study including 101 patients, 83.2% of patients receiving Mohs micrographic surgery for skin cancer underestimated their scar size. Scars were a median of 2.2 times larger than patients expected, compared with 1.1 times larger for physicians’ estimates.

**Meaning:**

These findings suggest that scars from Mohs micrographic surgery for facial skin cancers may be longer than patients expect.

## Teaser

This cross-sectional study compares preoperative estimates of scar length by patients and surgeons with the scar length after Mohs micrographic surgery for facial skin cancers.

## Introduction

Mohs micrographic surgery (MMS) is touted for its high cure rate and ability to spare healthy tissue.^1^ Patients focused on tissue sparing may have unrealistic expectations for small scars after MMS. Patients with facial skin cancers place high importance on a normal appearance after surgery.^2,3,4^ Scars following skin cancer surgery have been shown to diminish patients’ quality of life,^2,3,4^ and larger-than-expected scars may increase patient distress.^5^

Setting realistic expectations about scarring after surgery for facial skin cancers may increase patient satisfaction and decrease litigation risk. One of the most common reasons for litigation against dermatologists is failure to instruct or communicate with patients.^6^ Aligning expectations about scarring may be especially important for surgeons who perform MMS, because one of the most common reasons that patients sue these surgeons is dissatisfaction with the functional and cosmetic outcome.^7^

Numerous studies from other surgical specialties have shown that patients are dissatisfied when their expectations do not match the actual outcome.^5,8,9,10,11,12,13,14,15,16,17,18^ Few studies have examined patient expectations for scarring after skin cancer surgery.^5^ The primary objective of this study was to compare patients’ and surgeons’ preoperative estimations of scar length with actual scar length after MMS for facial skin cancers. A secondary objective was to assess whether preoperative consultation with a surgeon who performs MMS, a personal history of undergoing MMS, or patient-directed research were associated with more accurate estimates of scar length. Data from this study may help surgeons align patient expectations before surgery of facial skin cancers.

## Methods

A prospective, observational, cross-sectional study was performed on patients older than 18 years presenting for MMS for treatment of facial skin cancers at the University of Pennsylvania between December 1, 2017, and February 28, 2018. Skin cancers were included if they were located on the forehead, temples, cheeks, nose, and perioral or periorbital areas. Skin cancers of the ears, neck, scalp, and the remainder of the body were excluded. Patients undergoing surgery for multiple separate skin cancers were enrolled for only 1 skin cancer. The University of Pennsylvania Institutional Review Board approved the study, and informed consent was obtained orally from study participants. Patients enrolled in the study did not receive financial compensation. This study followed the Strengthening the Reporting of Observational Studies in Epidemiology (STROBE) reporting guideline for cross-sectional studies.

Prior to MMS, one of us (W.C.F.) asked consenting patients to look in a mirror and use a washable marker to draw on their face the scar they expected after MMS. The skin markings were photographed and the length of a single line or longest dimension of a nonlinear marking was measured. The markings were wiped clean before the surgeon performing the procedure met the patient.

The surgeon independently drew on the patient’s skin the expected size and shape of the scar after surgery. Again, the skin markings were photographed, and the length of a single line or longest dimension of a nonlinear marking was measured.

Reconstruction was performed after confirming clear microscopic margins with MMS. The length of a linear closure was recorded or the longest dimension of a nonlinear closure (eg, flap or graft) was recorded immediately after closure was completed.

The longest dimension (henceforth *length*) of the preoperative estimated scar from the patients and surgeons was considered correct if it measured within 75% and 125% of the length of the postoperative scar. Preoperative skin markings less than 75% of the postoperative scar length were considered underestimations, and preoperative skin markings greater than 125% of the postoperative scar length were considered overestimations.

Patients completed a survey about the education or experiences that could influence their scar estimates: what resources they consulted to learn about MMS, whether they had previously undergone MMS for another skin cancer, and whether they had a consultation with the surgeon who was to perform the procedure before the day of their surgery. Patients self-reported sex and race/ethnicity.

### Statistical Analysis

A target sample size of 100 was chosen for this observational study. The Fisher exact test was used to compare the proportion of correct length, as well as overestimates and underestimates of length among patients and physicians, and to compare correctness in subgroups of patients. Actual scar to estimation length ratios were compared between patients and physicians using a Wilcoxon signed rank test and across subgroups using a Mann-Whitney test. Medians and interquartile ranges (IQRs) were used as a measure of central tendency and distribution. A linear regression model constructed using forward selection with a *P* value cutoff level of .10 was used to assess factors in the accuracy of patients’ estimations, which was measured as the actual scar to estimation length ratio. A *P* value of .05 used with a 2-tailed *t* test was considered statistically significant for all statistical tests of the hypothesis listed above. Stata, version 15 (StataCorp) was used for statistical analysis.

## Results

A total of 101 patients with 101 tumors enrolled in the study; 57 patients (56.4%) were aged 65 years or older, 99 were of white, non-Hispanic race/ethnicity (98.0%), and 57 were men (56.4%). Table 1 summarizes patient demographics and case characteristics. Patients preoperatively drew the expected scar in all 101 tumors. The surgeon preoperatively drew the expected scar in 86 tumors. The surgeon’s estimates were not recorded in 15 patients because either the surgeon did not mark the estimated scar or the research assistant did not have a chance to measure the estimated scar length before surgery. The median postoperative scar length did not differ significantly between the cases with and without the surgeon’s estimate.

**Table 1.** Demographic and Health Characteristics of the Study Cohort

Characteristic	No. (%)
All cases	101 (100)
Age, y	
<65	44 (43.6)
≥65	57 (56.4)
Race/ethnicity	
White, non-Hispanic	99 (98.0)
White, Hispanic	1 (1.0)
Not specified	1 (1.0)
Sex	
Female	42 (41.6)
Male	57 (56.4)
Not specified	2 (2.0)
Smoking status	
Smoker	10 (9.9)
Nonsmoker	91 (90.1)
History of Mohs micrographic surgery	
Yes	50 (49.5)
No	51 (50.5)
Preoperative consultation	
Yes	33 (32.7)
No	68 (67.3)
Use of resources	
Yes	65 (64.4)
No	36 (35.6)
Diagnosis	
Basal cell carcinoma	42 (41.6)
Squamous cell carcinoma	32 (31.7)
Melanoma or melanoma in situ	21 (20.8)
Other	6 (5.9)
Reconstruction type	
Linear closure	56 (55.4)
Local flap	21 (20.8)
Interpolation flap	7 (6.9)
Graft	8 (7.9)
Secondary intention	4 (4.0)
Wedge repair	2 (2.0)
Other or not specified	3 (3.0)
No. of stages	
1	86 (85.1)
≥2	15 (14.9)

Table 2 compares the patients’ and surgeons’ preoperative scar length estimates with the actual postoperative scar length. The median postoperative length of the MMS scars was 47 mm (IQR, 32-70 mm). Postoperative scars were a median 2.2 (IQR, 1.5-3.6) times longer than patients expected, compared with 1.1 (IQR, 1.0-1.2) times longer than the surgeons expected (*P* < .001).

**Table 2.** Scar Length by Actual, Patient, and Surgeon Estimates

Parameter	Median (IQR) [Range]
Postoperative scar length, mm	47 (32-70) [9-180]
Patient preoperative length estimate, mm	20 (13-30) [4-80]
Surgeon preoperative length estimate, mm	41 (30-65) [10-130]
Ratio of actual length to patient length estimate	2.2 (1.5-3.6) [0.1-16.2]
Ratio of actual length to surgeon length estimate	1.1 (1.0-1.2) [0.2-6.2]

Abbreviation: IQR, interquartile range.

**Figure 1.**
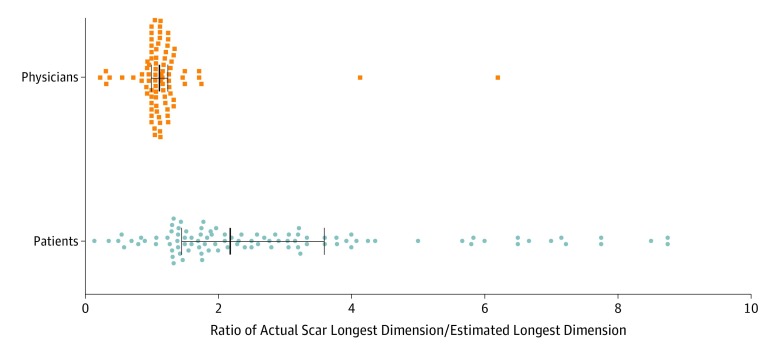
Ratios of Actual Postoperative Scar Length After Mohs Micrographic Surgery to Surgeon- and Patient-Estimated Preoperative Scar Length Each dot represents the ratio of the actual postoperative to estimated preoperative scar length. Center lines and error bars indicate median values and interquartile ranges, respectively. The median actual scar length was 2.2 times larger than the patients’ estimates. Physicians estimated more accurately, with a 1.1 ratio of median actual scar length to estimated scar length.

Figure 1 illustrates the ratio of the actual postoperative scar length to the estimated preoperative scar length for both patients and surgeons. Compared with the surgeons, patients estimated that scars would be much shorter than the postoperative scar, and patient expectations of scar length varied more than those of the surgeons (Figure 1 and Table 2).

**Figure 2.**
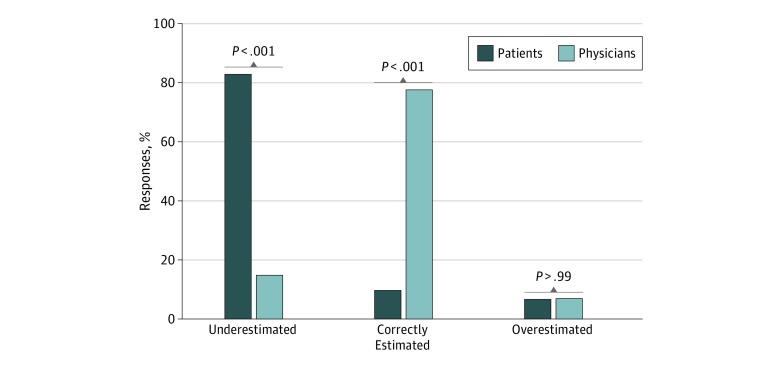
Comparison of Surgeon and Patient Scar Estimates Patients were significantly more likely to underestimate scar length than surgeons performing Mohs micrographic surgery and significantly less likely to correctly estimate scar length. Overestimation of the scar length was equally uncommon among patients and physicians.

Figure 2 shows the proportions of patients and physicians who underestimated, correctly estimated, or overestimated the scar length. Patients were significantly more likely than surgeons to underestimate scar length (84 of 101 [83.2%] vs 13 of 86 [15.1%]; *P* < .001). Surgeons were significantly more likely than patients to estimate correct scar length (67 [77.9%] vs 10 [9.9%]; *P* < .001). Patients and surgeons rarely overestimated scar length (Figure 2).

**Figure 3.**
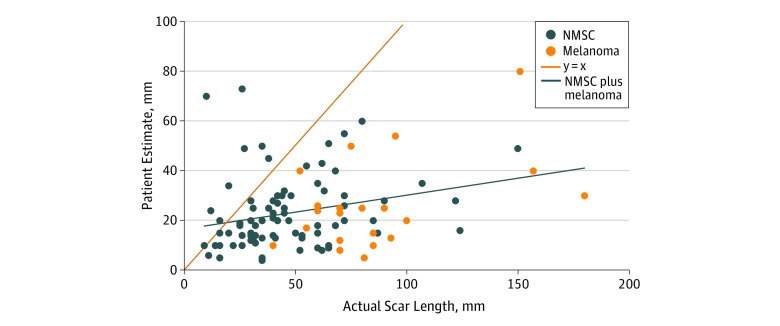
Actual Scar Length and Patient Estimated Scar Length The y = x line represents a perfectly accurate estimate. The points to the left of x = y represent patients who estimated a scar longer than the actual scar. The points to the right of x = y represent patients who estimated scars shorter than the actual scar. The line indicating nonmelanoma skin cancer (NMSC) plus melanoma suggests that patients underestimated the scar by a larger margin as the actual scar length increased.

Exploratory subgroup analysis showed that diagnosis of melanoma was associated with longer postoperative scars than nonmelanoma skin cancer diagnosis (median, 80 mm; IQR, 70-93 mm for melanomas vs 40.5 mm; IQR, 30-61 mm for nonmelanoma skin cancer; *P* < .001). Patients with melanoma underestimated the scar by a larger margin than patients with nonmelanoma skin cancer (actual length to estimated length ratio, 3.6; IQR, 2.5-5.8 for melanomas vs 1.9; IQR, 1.4-3.2 for nonmelanoma skin cancer; *P* < .001). Figure 3 illustrates that patients tended to underestimate scar length by a greater margin as the length of the actual scar increased.

Education through preoperative consultations, patient-initiated research about MMS, or past experience with MMS for another skin cancer did not significantly improve the accuracy of patients’ preoperative estimations of scar length. Reconstruction type and the number of Mohs stages were not associated with the accuracy of patient or physician estimations, nor were other patient demographic characteristics. Individual surgeons did not vary in the accuracy of their estimates of scar length.

## Discussion

This study used a novel method to evaluate 3 key findings about patient and surgeon preoperative estimates of scar length following MMS for facial skin cancers. First, most patients expected scars less than half as long as the actual postoperative scar. Second, surgeons who performed the procedure usually estimated scar length accurately, and if they underestimated scar length, the margin of difference from the actual scar is small. Third, traditional methods of patient education, such as preoperative consultation or web-based information, did not appear to improve the accuracy of patient expectations. These findings may help surgeons devise effective strategies to align patient expectations about scars resulting from MMS.

Most patients in this cohort (83.2%) substantially underestimated the length of their postoperative scars (Figure 2), and patient estimations varied widely (Figure 1 and Table 2), with patients tending to underestimate scar length by a greater margin as the actual scar length increased (Figure 3). Although this study did not associate patient expectations with postoperative satisfaction, unrealistic expectations may increase the risk for litigation against surgeons who perform MMS and may decrease satisfaction with outcomes, as has been shown in other surgical specialties.^5,8,9,10,11,12,13,14,15,16,17,18^

In comparison with patients, surgeons accurately estimated the scar size in this study and infrequently underestimated the size (Table 2, Figure 1, and Figure 2). These findings suggest that surgeons who perform MMS can use their accurate estimations to set realistic expectations that may improve patient satisfaction with scar length.^8,12,19^ Novel strategies for preoperative education may be important because patient-initiated research on MMS (through websites, pamphlets, or speaking with friends or family), a history of MMS, or preoperative consultation visits did not improve the accuracy of patient expectations for scar length. Other studies have demonstrated mixed outcomes with aligning patient expectations using printed information^20^ or other modalities, such as written quizzes.^12^

### Limitations

This study has limitations. To our knowledge, the method of assessing expectations of scar length by having patients draw directly on their skin has not been tested. We chose this method because, in our experience, patients frequently express surprise after the surgeon performing MMS draws the anticipated scar on their faces before surgery. Other studies have measured patient expectations with different methods, such as drawing the expected scar on a diagram of the human body and with postoperative survey questions asking patients if scars were not at all, somewhat, or a lot larger than they expected.^5^ Our method of having patients draw directly on their skin gives surgeons a reference for comparison and education when they draw a more accurate estimation of the anticipated scar.

## Conclusions

This study highlights that patients might expect unrealistically small scars after MMS for facial skin cancer and the surgeons accurately estimate the length of most surgical scars and have an opportunity to set realistic patient expectations about scar length before surgery. Existing literature suggests an association between expectation fulfillment and patient satisfaction.^5,8,9,10,11,12,13,14,15,16,17,18^ Further investigation is needed to determine whether setting realistic expectations prior to MMS can improve patient satisfaction.

## References

[1] Miller CJ, Neuhaus IM, Sobanko JF, Veledar E, Alam M Accuracy and completeness of patient information in organic World-Wide Web search for Mohs surgery: a prospective cross-sectional multirater study using consensus criteria. Dermatol Surg. 2013;39(11):-. doi:10.1111/dsu.12344 24118592

[2] Sobanko JF, Sarwer DB, Zvargulis Z, Miller CJ Importance of physical appearance in patients with skin cancer. Dermatol Surg. 2015;41(2):183-188. doi:10.1097/DSS.0000000000000253 25654193

[3] Zhang J, Miller CJ, O’Malley V, Patient and physician assessment of surgical scars a systematic review. JAMA Facial Plast Surg. 2018;20(4):314-323. doi:10.1001/jamafacial.2017.2314 29392275

[4] Zhang J, Miller CJ, O’Malley V, Etzkorn JR, Shin TM, Sobanko JF Patient quality of life fluctuates before and after Mohs micrographic surgery: a longitudinal assessment of the patient experience. J Am Acad Dermatol. 2018;78(6):1060-1067. doi:10.1016/j.jaad.2018.02.065 29518455

[5] Cassileth BR, Lusk EJ, Tenaglia AN Patients’ perceptions of the cosmetic impact of melanoma resection. Plast Reconstr Surg. 1983;71(1):73-75. doi:10.1097/00006534-198301000-00016 6849025

[6] Moshell AN, Parikh PD, Oetgen WJ Characteristics of medical professional liability claims against dermatologists: data from 2704 closed claims in a voluntary registry. J Am Acad Dermatol. 2012;66(1):78-85. doi:10.1016/j.jaad.2010.12.003 21757256

[7] Perlis CS, Campbell RM, Perlis RH, Malik M, Dufresne RG Jr Incidence of and risk factors for medical malpractice lawsuits among Mohs surgeons. Dermatol Surg. 2006;32(1):79-83. doi:10.1097/00042728-200601000-00016 16393602

[8] Hageman MGJS, Briët JP, Bossen JK, Blok RD, Ring DC, Vranceanu AM Do previsit expectations correlate with satisfaction of new patients presenting for evaluation with an orthopaedic surgical practice? Clin Orthop Relat Res. 2015;473(2):716-721. doi:10.1007/s11999-014-3970-6 25269531 PMC4294929

[9] Williams S, Weinman J, Dale J, Newman S Patient expectations: what do primary care patients want from the GP and how far does meeting expectations affect patient satisfaction? Fam Pract. 1995;12(2):193-201. doi:10.1093/fampra/12.2.193 7589944

[10] Bowling A, Rowe G, McKee M Patients’ experiences of their healthcare in relation to their expectations and satisfaction: a population survey. J R Soc Med. 2013;106(4):143-149. doi:10.1258/jrsm.2012.120147 23564898 PMC3618164

[11] Kohl E, Meierhöfer J, Koller M, Fractional carbon dioxide laser resurfacing of rhytides and photoaged skin—a prospective clinical study on patient expectation and satisfaction. Lasers Surg Med. 2015;47(2):111-119. doi:10.1002/lsm.22326 25652114

[12] Rossi MJ, Brand JC, Provencher MT, Lubowitz JH The expectation game: patient comprehension is a determinant of outcome. Arthroscopy. 2015;31(12):2283-2284. doi:10.1016/j.arthro.2015.09.005 26652147

[13] Noble PC, Conditt MA, Cook KF, Mathis KB The John Insall Award: patient expectations affect satisfaction with total knee arthroplasty. Clin Orthop Relat Res. 2006;452(452):35-43. doi:10.1097/01.blo.0000238825.63648.1e 16967035

[14] Halawi MJ, Vovos TJ, Green CL, Wellman SS, Attarian DE, Bolognesi MP Patient expectation is the most important predictor of discharge destination after primary total joint arthroplasty. J Arthroplasty. 2015;30(4):539-542. doi:10.1016/j.arth.2014.10.031 25468779

[15] Halawi MJ, Vovos TJ, Green CL, Wellman SS, Attarian DE, Bolognesi MP Preoperative pain level and patient expectation predict hospital length of stay after total hip arthroplasty. J Arthroplasty. 2015;30(4):555-558. doi:10.1016/j.arth.2014.10.033 25433645

[16] Conner-Spady BL, Sanmartin C, Johnston GH, McGurran JJ, Kehler M, Noseworthy TW The importance of patient expectations as a determinant of satisfaction with waiting times for hip and knee replacement surgery. Health Policy. 2011;101(3):245-252. doi:10.1016/j.healthpol.2011.05.011 21680042

[17] Waljee J, McGlinn EP, Sears ED, Chung KC Patient expectations and patient-reported outcomes in surgery: a systematic review. Surgery. 2014;155(5):799-808. doi:10.1016/j.surg.2013.12.015 24787107 PMC4170731

[18] Dizon M, Linos E, Arron ST, Comparisons of patients’ satisfaction should take expectations into account. Br J Dermatol. 2017;176(1):252-254. doi:10.1111/bjd.14755 27203511

[19] Prakash B Patient satisfaction. J Cutan Aesthet Surg. 2010;3(3):151-155. doi:10.4103/0974-2077.74491 21430827 PMC3047732

[20] Nasr IH, Sayers M, Newton T Do patient information leaflets affect patients’ expectation of orthodontic treatment? a randomized controlled trial. J Orthod. 2011;38(4):257-268. doi:10.1179/14653121141614 22156181

